# FASTING IN ELECTIVE SURGICAL PATIENTS: COMPARISON AMONG THE TIME PRESCRIBED, PERFORMED AND RECOMMENDED ON PERIOPERATIVE CARE PROTOCOLS

**DOI:** 10.1590/S0102-6720201500040008

**Published:** 2015

**Authors:** Saionara Cristina FRANCISCO, Sandra Teixeira BATISTA, Geórgia das Graças PENA

**Affiliations:** 1 Odilon Behrens Municipal Hospital, Belo Horizonte, MG; 2 Nutrition Course, Faculty of Medicine, Federal University of Uberlândia, Uberlândia, MG, Brazil

**Keywords:** Fasting, Perioperative care, Elective surgical procedures, Nutritional support

## Abstract

**Background:**

Prolonged preoperative fasting may impair nutritional status of the patient and their recovery. In contrast, some studies show that fasting abbreviation can improve the response to trauma and decrease the length of hospital stay.

**Aim:**

Investigate whether the prescribed perioperative fasting time and practiced by patients is in compliance with current multimodal protocols and identify the main factors associated.

**Methods:**

Cross-sectional study with 65 patients undergoing elective surgery of the digestive tract or abdominal wall. We investigated the fasting time in the perioperative period, hunger and thirst reports, physical status, diabetes diagnosis, type of surgery and anesthesia.

**Results:**

The patients were between 19 and 87 years, mostly female (73.8%). The most performed procedure was cholecystectomy (47.69%) and general anesthesia the most used (89.23%). The most common approach was to start fasting from midnight for liquids and solids, and most of the patients received grade II (64.6%) to the physical state. The real fasting average time was 16 h (9.5-41.58) was higher than prescribed (11 h, 6.58 -26.75). The patients submitted to surgery in the afternoon were in more fasting time than those who did in the morning (p<0.001). The intensity of hunger and thirst increased in postoperative fasting period (p=0.010 and 0.027). The average period of postoperative fasting was 18.25 h (3.33-91.83) and only 23.07% restarted feeding on the same day.

**Conclusion:**

Patients were fasted for prolonged time, higher even than the prescribed time and intensity of the signs of discomfort such as hunger and thirst increased over time. To better recovery and the patient's well-being, it is necessary to establish a preoperative fasting abbreviation protocol.

## Abstract (pt)

**Racional:**

O jejum perioperatório prolongado pode prejudicar estado nutricional do paciente e sua recuperação. Em contrapartida, estudos mostram que abreviação do jejum pode melhorar a resposta ao trauma e diminuir o tempo de internação.

**Objetivo:**

Investigar se o tempo de jejum perioperatório prescrito e praticado pelos pacientes se encontra em conformidade com os protocolos multimodais atuais e identificar os principais fatores associados.

**Métodos:**

Estudo transversal com 65 pacientes, submetidos às operações eletivas do aparelho digestivo ou parede abdominal. Foi investigado o tempo de jejum no período perioperatório, relatos de fome e sede, classificação do estado físico, diagnóstico de diabete, tipo de operação e de anestesia.

**Resultados:**

Os pacientes tinham entre 19 e 87 anos, sendo a maioria do sexo feminino (73,8%). O procedimento mais realizado foi colecistectomia (47,69%) e a anestesia geral a mais usada (89,23%). A orientação mais frequente foi jejum a partir de meia noite para líquidos e sólidos e a maior parte dos pacientes recebeu classificação II (64,6%) para o estado físico. O tempo médio de jejum real foi 16 h (9,5-41,58) sendo maior que o prescrito (11 h; 6,58-26,75) com p<0,001. Aqueles que realizaram a operação no período da tarde ficaram mais tempo de jejum do que aqueles do período matutino (p<0,001). A intensidade de fome e sede aumentou no período de jejum pós-operatório (p=0,010 e 0,027). O período de jejum médio pós-operatório foi 18,25 h (3,33-91,83) e apenas 23,07% reiniciou a alimentação no mesmo dia.

**Conclusão:**

Os pacientes permaneceram em jejum por tempo prolongando, ainda maior que o tempo prescrito e a intensidade dos sinais de desconforto como fome e sede aumentaram ao longo do tempo. Para melhor recuperação e bem-estar do paciente, faz-se necessário instituir um protocolo de abreviação do jejum perioperatório.

## INTRODUCTION

Elective surgery is a trauma which occurs catabolic process and changes in immune and inflammatory systems, in order to restore homeostasis and repair the damages tissues^9^^,^ 21 ^,^ 28.

Prolonged preoperative fasting with hypercatabolism caused by metabolic stress of the surgical trauma induces damage in the nutritional status or exacerbation of possible previous malnutrition. Besides that, this process could increase the insulin resistance, risk of infection, decrease intestinal integrity or impairment of the healing process and could prolong the hospital stay^4^^,^ 25 ^,^ 29 ^,^ 30.

The prescription of preoperative fasting of the 8 to 12 h was first published by Mendelson in 1946. This procedure was adopted empirically and it is practiced until lately, even in the era of evidence-based medicine^3^^,^ 24.

The major concern is the risk of aspiration associated with anesthesia. However, recent studies showed that there is not this risk to reduce the preoperative fasting. The American Society of Anesthesiologists (ASA) recommends in their practical guide, (published in 1999 and revised in 2011) that the preoperative fasting should be 2 h to zero of liquid diet enriched with carbohydrates without waste, with or without nutritional content, for example water, tea, coffee juices fruits without pulp and beverages rich in carbohydrates before elective procedures requiring general anesthesia or local sedation. For solids, the recommendation is 6 h for snacks and 8 h for meals^10^^,^ 23.

The liquid diet enriched with carbohydrates in 2 h before surgical procedures may provide benefits such as prevention of immunosuppression, reduced risk of infectious complications, earlier return of bowel function, decreased sensation of thirst, hunger, nausea and vomiting, insulin resistance attenuation and help to maintain muscle strength, decreased hospital stay and improves the response to trauma^2^^,^ 5 ^,^ 27.

Similarly, the traditional conduct postoperative only permit the return to diet after the clinical signs of peristalsis such as, elimination of flatus, appearance of abdominal sounds or evacuation characterizing the end of postoperative ileus. These practice increase the postoperative fasting for on average up to five^1^. 

The reduction of fasting helps faster returns gastrointestinal function, better metabolic recovery, reduce hospital stay, costs and the complication rates^11^^,^ 12 ^,^ 19 ^,^ 30. Researchers and physicians from of five universities of the north of Europe developed in 2001 the ERAS (Enhanced Recovery After Surgery) protocol in order to decreased the surgical complications^15^^,^ 16 ^,^ 20. 

In this sense, the Department of Surgery of Faculty of Medical Science Clinic of the Federal University of Mato Grosso, Brazil, developing in 2005 the ACERTO (Aceleração da Recuperação Total Pós-Operatória) to adjust the changes proposed by ERAS project to the brazilian reality. Their protocols include, among other things, the reduction of preoperative fasting, the use of liquid diet enriched with carbohydrate up to 2 h before surgery and release of diet on the first day postoperative (6-24 h) for the most surgical procedures^12^.

Thus, the aim of study was to investigate the perioperative fasting and associated factors in elective surgical patients comparing the prescribed, performed and recommended times in a public hospital in Minas Gerais, Brazil.

## METHODS

Cross-sectional study was conducted in elective surgical patients in the gastrointestinal tract between July and November, 2014. The project was approved by the Ethics Committee of the evaluated Hospital (CAAE: 31943814.3.0000.5129). The patients with 18 years and moreover were include, while patients diagnosed with severe obesity, gastroesophageal reflux, pyloric stenosis syndrome or obstruction of the gastrointestinal tract were not included, following the criteria of the protocol. 

The convenience sample was used and it was based on the average number of surgical procedures per month considering the inclusion or exclusion criteria. Among the selected patients were included those who agreed to participate and signed the consent form.

The time of the last intake of solids and liquids in the preoperative period and the use at least 400 ml of beverages enriched with 12.5% carbohydrates were asked as recommended in perioperative care protocol^1^. 

The preoperative fasting was considered the time between the last intake of solids and liquids and the anesthetic induction. The liquids fasting without nutritional content was calculated apart. The postoperative fasting time was considered from of the end of surgical procedure and the first liquids or solid ingestion. To compare the differences of the scheduled fasting and what the really fasting and the difference of these informations, the patients were asked to questions about what and who was the fasting request.

The thirsty or hungry symptoms were asked during the fasting in the pre and postoperative. To evaluate the intensity, was used a numerical verbal scale (NVS) with 11 points, numbered from 0 to 10, where zero was the absence, five as moderate and ten as intensity of these symptoms.

Age, gender, diabetes, surgical procedure, prescribe liquid and solid fasting, technique and induction anesthetic and the ASA (American Society of Anesthesiologists) level was collected from records. To check delays were observe the scheduled time for the procedures in the note of the operations in the hospital room.

Analyzes were performed by Statistical Package for the Social Sciences (SPSS) version 22.0. First of all, was observed the distribution of the variables by Kolmogorov-Smirnov test. The parametric and non-parametric tests were used depending of their distribution. Only the surgical procedure time and prescribe fasting to liquids showed normal distribution. The median (minimum-maximum) and frequencies were used to describe variables. The U de Mann-Whitney test was used to compare groups and Kruskal Wallis test to three or more groups to independent variables. Lastly, the paired Wilcoxon test was used to compare dependent groups. Was used the significance level of p-value< 0.05.

## RESULTS

Sixty-five patients were include and six excluded because incomplete data (9.2%). The majority were female (73.8%) and between 37 and 53 years (47.69%) raging 19 and 87 years. The surgical procedure more frequency was the laparoscopic cholecystectomy (47.69%). In seven patients (10.77%), the general anesthesia was not used in the surgical procedure. The frequency the ASA I was 29.23% and ASA II 64.61% levels. All patients were feeding by oral. Thirteen patients (20%) were diabetic record (Table 1).

The most of surgical procedures occurred in the (52.3%) and 38.4% were defined for the first schedule. The last meal was made out of home in the 40% of the patients because they were admitted in the same morning of the surgical procedure.

When the surgical procedure happened in the afternoon the fasting time was increased (19 h; CI=9.75 to 41.58 h) compared with the morning surgeries (12.25 h; CI=50 to 27.42 h), p<0.0001. Until the previous day, 40% of patients with the procedures in the afternoon not have scheduled and they were waiting a vacancy in operating room. Three patients had their procedures rescheduled because emergency situations.

During the preoperative period, none patient consumed beverages enriched in carbohydrates. The prescribe fasting for the afternoon procedures was start at midnight (83.07%) for both liquid and solid. Among patients who undergo the afternoon procedures, only three (4.61%) were advised to start fasting for liquids and solids at 8 h in the morning. The others situations were diverse and for three patients was not mentioned fasting for liquids.

**TABLE 1:** - Description of the variables

**Variables**	**n**	**%**
Gender		
Female	48	73.84
Male	17	26.15
Age (years)		
19 - 36	19	29.23
37 - 53	31	47.69
54 - 70	11	16.92
71 - 87	4	6.15
ASA level		
I	19	29.23
II	42	64.61
III	4	6.15
Surgical procedure		
Laparoscopic cholecystectomy	31	47.69
Papillotomy	7	10.76
Liver abscess drainage	7	10.76
Enterostomy closing / colostomy	7	10.76
Others	13	20.0
Type of anesthesia		
General	58	89.23
Spinal anesthesia	6	9.23
Local	1	1.54
Diabetes		
Yes	13	20.0
No	52	80.0

For the prescribed fasting, the average of the elapsed time between the prescribed and the beginning of the surgical procedure should have been 11 h (6.58-26.75 h) for liquids and solids. However, the time of fasting actually practiced by patients was 16 h (9.5-41.58 h) and was greater than the instructed time (p<0.0001) showed in the Table 2. The prescribed and practiced fasting period for liquids was different by patients (p<0.0001).

**TABLE 2:** - Median fasting time in the preoperative period

	**Liquids**		**Solids**			
	**Median**	**Variation**	p-value*	**Median**	**Variation**	p-value*
Preoperative fasting (hours)						
Prescribe	10.41	(2.75 to 17)	<0.0001	11	(6.58 to 26.75)	<0.0001
Practice	15.50	(1.50 to 25)		16	(9.5 to 41.58)	

*Wilcoxon test

Prescribed preoperative fasting for solids and liquids were higher in diabetic, if patients had followed the recommendation of the instructors, was statistically higher among diabetics (p=0.013 and 0.007). Between than the majority was submitted to procedures in the afternoon (60%) and just one patient was oriented to start the fasting before midnight. It was not observe differences between fasting and ASA level (0.067 and 0.805), type of procedure (p=0.613 and 0.916) and age (p=0.073 and 0.670) for solids and liquids, respectively. Regarding the practiced fasting for solids and liquids, the differences were observed by only ASA level (p=0.018). The ASA III patients had higher fasting that ASA I and II (post hoc test; 0.014 and 0.043, respectively).

Thirsty was the symptom more frequently during the preoperative fasting (69.23%); however, and 35.38% reported hunger in this period. The fasting was not associated with thirsty (p=0.296) neither hunger (p=0.956). These symptoms were also similar between gender (0.07 and 0.641, respectively). The score of verbal analog scale (NVS) in preoperative, was 2 for thirsty (1ºquintil=0 and 3rd quintile=9) and 0 for hunger (1ºquintil=0 and 3rd quintile=5). The intensity of hunger and thirst was not different by afternoon programmed procedures and these ones had obviously higher fasting time (p=0.859 and 0.856). The intensity of hunger or thirst were not associated with gender (p=0.055 and 0.073).

The mean fasting period of postoperative was 18.25 h (3.33-98.83 h) for solids and 17.41 h (2.50-69.58 h) for liquids, (p=0.337). The frequency of returned feed in the postoperative was 76.92%. Just 15 patients had the first meal in the same day of the surgical procedure 23,07%. Of the 48 patients that had procedures related by biliary system or hernias, just 13 (27.08%) had prescribed of diet in until 12 h as recommended by protocols, showing a median of 17.45 h (3.33-91.83 h). The patients submitted to cholecystectomy showed lower postoperative fasting when compare on the other (p=0.003).

**TABLE 3:** - Median fasting in the postoperative

	**Liquid diet**	**Solid diet**						
	**n**	**%**	**Median**	Variation*	**n**	**%**	**Median**	Variation*
Postoperative fasting (h)								
1º POD	50	76,92	15	(2,50-24)	50	76,9	15,5	(3,33-24)
Surgical procedure day	16	24,61	5,87	(2,50-11)	15	23,07	6	(3,33-11)
2º POD	8	12,30	28,29	(26,50-45,66)	8	12,30	27,65	(26,55-45,66)
Since 3th POD	7	10,76	66,08	(49,01-69,58)	7	10,76	68,55	(49,01-98,83)
Total	65	100	17,41	(2,5-69,58)	65	100	18,5	(3,33-98,83)

POD=postoperative day; *variation in minimum to maximum

**FIGURE 1:**
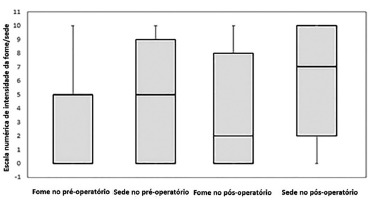
- Numerical verbal scale (NVS) to hunger and thirsty in pre and postoperative.

The frequency of thirsty in the postoperative fasting was 73.85% and hunger was 52.31% and they were not associated with the fasting duration (p=0,247 e 0,753, respectively). The median of NVS to hunger was 2 (1^st^quintil=0 e 3^th^ quintil=8) and thirsty was 7 (1^st^quintil=2 e 3^th^ quintil=10). The symptoms of hunger and thirsty were higher in the postoperative than preoperative periods (Wilcoxon test; p=0.010 and 0.027, respectively, Figure 1). 

The mean of the postoperative hospital stay was two days (1-18) and 17% remain in hospital for four days or more. The hospital stay after surgery not showed difference with preoperative fasting duration, p=0.128. Lastly, the mean time of surgical procedures was 3.2 ±1.68 h.

## DISCUSSION

This study showed that the mean fasting time is higher than prescribed. Besides, the afternoon patients were submitted a higher fasting time. The hunger and thirsty became higher in this same period. The perioperative care routines follow the traditional model and became more distant of the evidence-based medicine and prolong the fasting beside the prescribed and increased the discomfort of patients.

The fasting time to liquids and solids were very higher that ASA recommended. The result is the double of maximum value recommended to solids and higher almost eight times the recommendation to liquids. This result is similar to other studies done in hospitals that did not adhere to the multimodal protocols for perioperative care, as well as the present study.

Aguilar Nascimento *et al*. (2008)^13^ compared the clinical outcomes before and after implementation of the ACERTO protocol and found mean a fasting time of 16 h in preoperative before to deploy of protocol^3 ^in 308 patients included in the survey. Cestonaro *et al.* (2014)^8^ found 16.5 h of mean fasting time to solids and 15.75 h to liquids in a university public hospital in the South of Brazil.

It is important to note that the practice fasting was different of recommended. The reason for that is the belief that the higher fasting decreases the risk of complications. The patients reported that preferred to do the last meal earlier in preparation for the operation to be held the next day. The other reason is the hospital schedule in the food distribution because the last meal is offer by the hospital around of 21 h. The better routine in this sense is offer a beverage enriched with carbohydrates, 2 h before the surgical procedure.

The practice fasting higher that prescribed is not exclusive of hospitals that follow the traditional recommendations. A study published by Aguilar-Nascimento *et al.* in 16 hospitals in five regions of Brazil, found a median of 8 h (2-48h) in the group that follow the new recommendations and 13 h (6-21h) in group that used the traditional recommendations^8^^,^ 14. 

These findings suggest the persistent gap between the perception of the procedures adopted and the actually practiced. Even the prescribe fasting had been lower that followed by patients and it is distant that the ERAS and ACERTO protocols. The values found for liquid and solid fasting indicates there is still resistance to the new paradigms proposed by the current multimodal protocols, despite numerous studies that show the safety of abbreviated fasting. 

A systematic review with 38 randomized controlled studies concluded that there is no evidence to suggest that the shorter fasting increases the risk of aspiration, regurgitation during anesthesia or is related to increased morbidity^19^. Other study published by the Cochrane adds that doctors should be encouraged to re-evaluate and adjust the traditional policy of fasting^23^.

The guidelines for fasting were modifying over time and because the practicality of the rule "anything by mouth from midnight" was adopted for those with operation scheduled for the morning. It is common observe the same practice for the surgical procedures that happened in the afternoon. In this study, the patients that suffer afternoon procedures were fasted more than who did morning procedures. In addition to the practical sense, other factors contributes for the higher fasting time, for example, the absence of schedule time for surgical procedure (40%) and the delay time by emergency surgical procedures.

In this study, the recommendation was comply in a minority of the biliar and hernias procedures within the 6 to 12 h postoperative fasting time^1^. The delay of return to ingestion was associated the controversial and traditional conduct that consist in await the gastrointestinal sounds to ensure the return of peristalsis. 

Other care routine without scientific and safe evidences is related with digestive anastomoses, in which beliefs that bowel rest ensures adequate healing^12^^,^ 18. This practice could cause increased of inflammatory mediators, including insulin resistance, negative nitrogen balance, and this way, compromising the healing and increasing the risk of infections^2^^,^ 3 ^,^ 7 ^,^ 22 ^,^ 26.

On the other hand, the shorter fasting time in the first postoperative reduce the risk and bring benefits. A meta-analysis based on 15 randomized controlled studies involving 1240 patients showed that shorter fasting time could reduce the postoperative complications compared to the conduct traditional^17^.

More than half of patients reported moderated or intense thirsty and 30% hunger in postoperative period. Regarding the comparison of hunger and thirsty of the pre and postoperative period, these symptoms increasing significantly. Like shown before, none of the patients included in this study had the prescription of beverage enriched with carbohydrate. The discomfort has been associated as a predicted factor to undesirable results^8^. On the other hand, some studies showed that the offer of beverage enriched in carbohydrate in the preoperative period and the abbreviation of fasting time in the postoperative could reduce discomfort symptoms, including hunger and thirsty^26^. 

Hausel *et al.* (2001)^17^observed in 116 patients submitted to elective abdominal surgeries, lower scores on the visual analogue scale than the group that did the traditional fasting when ingested beverage enriched with carbohydrates 2 h before surgical procedures.

The fasting time was not associated with hunger and thirsty, i.e, patients who had higher preoperative fasting time no reported more hungry or thirsty than others with less fasting time. The insecurity about the time that would be actually carried out the surgical procedure and the anxiety, irritability and pain reported by patients, became thirsty and hunger a symptom in the background. However, more studies in this sense are necessary to explain these symptoms.

The fasting time was higher in the diabetics, probably because most of them performed the operation in the afternoon. This disease is not criteria to surgical procedures in the afternoon in the evaluated hospital and the instruction about the beginning of fasting time was similar to orientation given to patients no diabetic, suggesting that this result may have been occasional.

The mean time of hospital stay in the postoperative was 2 (1-18) days. In a study of Aguilar-Nascimento *et al.* 2 with patient submitted to cholecystectomy, it was observed that the length of hospital stay was one day on average in those who have reduced preoperative fasting time, which is shorter than that found in those who followed the traditional fasting. Another study showed that the accomplishment of postoperative care by the new protocols, including precocious feeding, also help to reduce the length of hospital stay in patients submitted colorectal procedures^6^. The reduction in length of stay and costs, enables higher turnover of beds and care of more patients.

## CONCLUSION

Patients fasted for a long time, even longer than the prescribed fasting time. The intensity of hunger and thirsty became higher with the time. Therefore, to better recovery and the patient's well-being, we reinforce that it is necessary to establish a preoperative fasting abbreviation protocol.
